# Dental implant in anterior mandible according to mandibular lingual foramens and lingual mucosal vessels: using fusion volumetric images from computed tomography and magnetic resonance imaging

**DOI:** 10.1186/s12903-023-03455-4

**Published:** 2023-10-08

**Authors:** Li Liu, Yijun Li, Yuchao Shi, Meng You, Jun Wang, Junichiro Sakamoto, Hu Wang

**Affiliations:** 1 State Key Laboratory of Oral Diseases, National Center for Stomatology, Department of orthodontics, West China Hospital of Stomatology, National Clinical Research Center for Oral Diseases, Sichuan University, Chengdu, 610041 Sichuan China; 2 State Key Laboratory of Oral Diseases, National Center for Stomatology, Department of Oral Medical Imaging, West China Hospital of Stomatology, National Clinical Research Center for Oral Diseases, Sichuan University, Chengdu, 610041 Sichuan China; 3 Dental Radiology and Radiation Oncology, Graduate School of Dentistry, Tokyo Medical and Dental University, Yushima 1- 5- 45, Bunkyo- ku, Tokyo, Japan

**Keywords:** Dental implants, CT, MRI, Imaging, Mandibular, Hemorrhage

## Abstract

**Background:**

Perforation of the lingual cortex while placing dental implants in the interforaminal region of the mandible can cause severe hemorrhage. The purpose of this study was to evaluate the features of mandibular lingual foraminals (MLFs) and locational relationship between them and lingual mucosal vessels (LMVs) by CT/MRI fusion volumetric images.

**Methods:**

37 images within complete anterior mandibular region using both MSCT and three-dimensional volumetric interpolated breath-hold examination (3D-VIBE) MRI were taken from our imaging archives. After exclusion of 11 for lesions or artifacts, 26 CT/MRI fusion volumetric images were included to evaluate the frequency, diameter, and position of MLFs. The anterior mandibular region was divided into 4 equal segments under each teeth, and 40 regions were got from C5 to D5. Furthermore, the positional relationship between MLFs and LMVs was analyzed in this coordinate system.

**Results:**

62 MLFs (73.81%) were located below the incisors, followed by premolars (21.43%) and canines (4.76%). Female bias, the mean diameter of the female was 0.08 mm while the male was 0.21 mm. The total number of LMVs was most distributed on lingual side of C1 and D1. According to Spearman’s correlation coefficient, the location of MLFs was related to LMVs. The MLFs in fourth segment of D1 were positively moderately correlated with LMVs in fourth segment of D4, while the MLFs in third segment of C1 showed a weak positive correlation with LMVs in third segment of D4.

**Conclusions:**

The features and the correlation between MLFs and LMVs in CT/MRI fusion volumetric images may offer reference to dentists when only MLFs can been seen on routine preoperative CT examination of implants.

**Trial registration:**

Retrospectively registered. (D2018-072)

## Background

Dental implants were widely used to replace missing teeth and were considered a safe surgical procedure for many years [1–3]. The interforaminal region of the mandible was the area located between the two mental foramina, traditionally regarded as a routine, relatively safe and uncomplicated site for implant placement [4, 5]. However, investigators in several studies reported incidences of hemorrhage and even life-threatening conditions such as airway obstruction happening in the restricted area [6–8].

Some investigators supported that canals occur in the anterior mandible and had been reported to cause significant bleeding if violated [9]. The mandibular lingual foramens (MLFs) were the potential anatomical complication for dental implant placement in the anterior mandible [10]. They were described as the small foramina in the midline of the mandibular lingual region. Cadaver studies had indicated that two main vessels named the sublingual artery (branch of the lingual artery) and the submental artery (branch of the facial artery) penetrate MLFs, which may be the cause of bleeding [11–13]. However, compared with MLFs, more researchers support that we should pay more attention to the perforations of the mandibular lingual cortex and respective vascular insult, which were the main reasons for bleeding. Tomljenovic overviewed 22 cases with life-threatening hemorrhage after a dental implant. All the cases detected the perforations of the mandibular lingual cortex [7]. Several case reports had pointed out that perforation of the lingual cortex while placing dental implants can cause severe hemorrhage [6, 8, 9, 14–22]. Since lingual mucosal vessels (LMVs) were scattered in the mandible, the vessels damage due to perforation of the lingual cortex was the most direct cause of bleeding, which indicated that the presence of vessels structures should be taken into consideration carefully before a dental implant.

Computed tomography (CT) was regarded as a routine and non-invasive investigation in dental implant. It was particularly important for presurgical planning in dental implants because it aided in the appropriate choice of implant size and help to avoid injury to critical structures [23]. Some studies demonstrated that CT can evaluate the frequency, diameter, position and direction of MLFs [24], but it can hardly evaluate the LMVs. The 3D volumetric interpolated breath-hold examination sequence of magnetic resonance imaging (3D-VIBE MRI) can be used to assess the LMV for a high signal-to-noise ratio and tiny isotropic voxel images [25], but less information for bony structure compared with CT. Hence, we tried to overcome such a potential challenge by using a fusion image technique. The purpose of this study was to evaluate the features of MLFs and locational relationship between MLFs and LMVs with the fusion volumetric image technique from CT and MRI. Although MRI is not used routinely in dentistry, this retrospective imaging studies may offer reference to dentists when only MLFs can been seen on routine preoperative CT examination of implants.

## Methods

### Patient selection and inclusion criteria

The study was approved by the institutional review board of Tokyo Medical and Dental University (D2018-072). In this retrospective study, cases with the complete anterior mandibular region examined by both 64-row multislice CT (MSCT) (Somatom Sensation 64 scanner, Siemens, Forchheim, Germany) and MR(Magnetom Spectra scanner, 3.0-T, 16-channel head and neck array coil, Siemens Healthcare, Erlangen, Germany)from January 2017 to April 2018 were collected from imaging archive. MSCT and MRI scanned in daily clinical protocols (MDCT: slice thickness: 0.6 mm; pitch: 0.6; tube voltage: 120 kV; tube current: 140 mAs; the field of view (FOV): 126 × 126 mm -157 × 157 mm) (MRI three-dimensional volumetric interpolated breath-hold examination (3D-VIBE): gadolinium (Gd)-enhanced: gadodiamide hydrate 0.2 ml/kg i.v.; repetition time/echo time: 13.7/3.9; flip angle: 20°; FOV: 150 × 150 mm; Matrix: 192 × 192 mm; Slice thickness: 0.8 mm with no intersection gap; Number of signals averaged: 1; The total scan time for 3D-VIBE imaging was 3 min and 35 s.). 37 images within complete anterior mandibular region using both MSCT and 3D-VIBE MRI were taken from our imaging archives. 9 cases with metallic artifacts or 2 cases with lesions in the mandibular anterior region were excluded. Twenty-six Japanese patients including 14 females and 12 males met the above criteria. The image diagnosis was inflammation (8 patients), cyst (10 patients), benign tumour (3 patients), malignant tumour (3 patients) and others (2 patients). Their age ranges from 11 to 81 years with an average age of 50.4 years.

### Fusion volumetric images

**Fig. 1:**
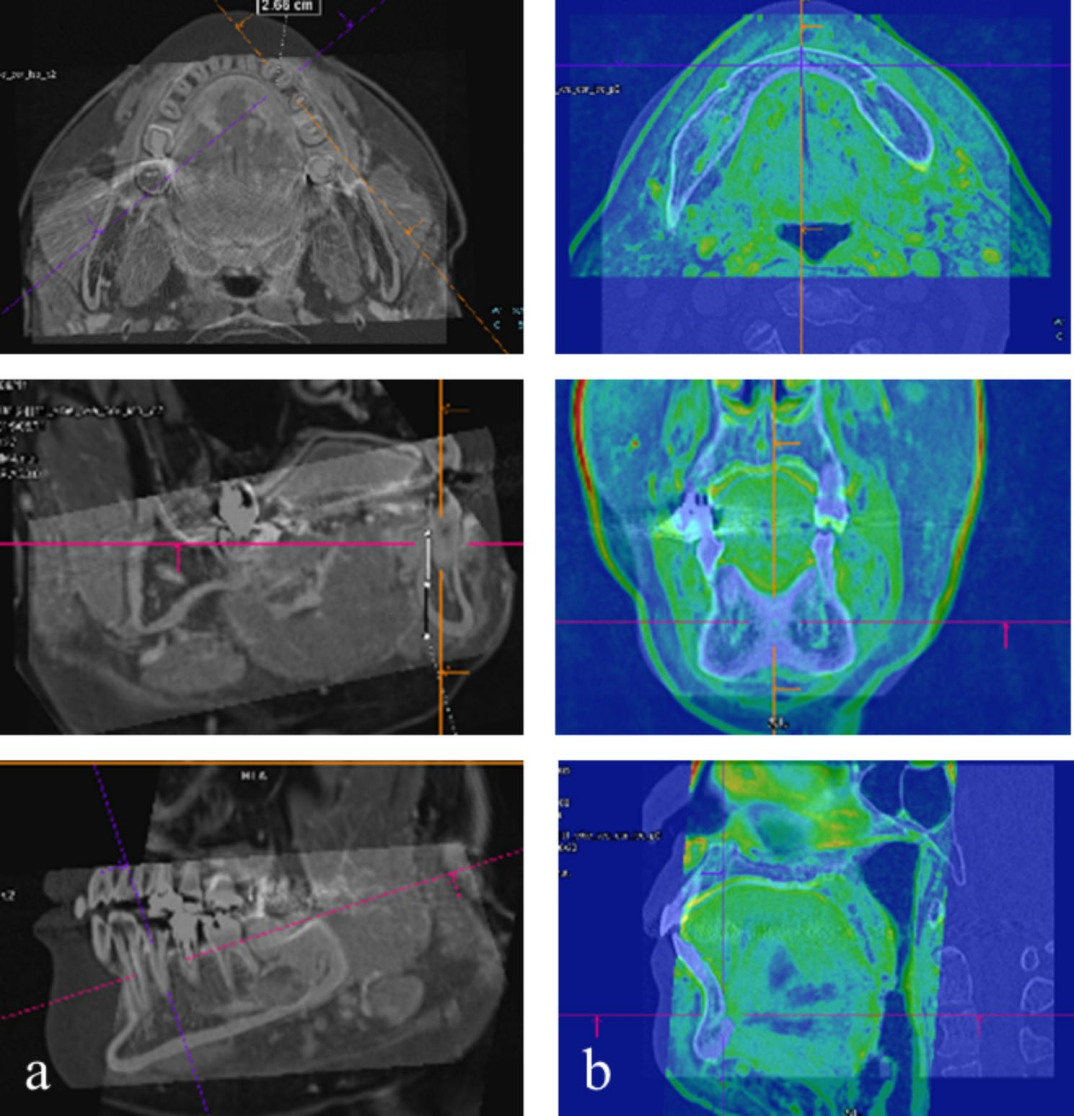
CT/MRI volumetric fusion images **a**. Fusion volumetric images before discoloration: CT/MRI volumetric fusion image was produced by two images of the mandible cortex completely overlapping **b**. Fusion volumetric images after discoloration

MSCT and MR images were transferred and fused on a syngo®.via VA20A workstation (Siemens). Both sets of images were multiplanarly reconstructed with the Multimodality Reading viewer. The fusion were taken by two oral and maxillofacial radiologists. The fusion was inaccurate sometimes when using automatic matching function. The images of the mandible cortex were differentiated between MSCT and MRI. So it was adjusted in detail manually by two images of the mandible cortex overlapping. Rarely, small disagreement between the two observers was resolved by discussion and a consensus was reached. So the final CT/MRI volumetric fusion image was produced by two images of the mandible cortex completely overlapping.(Fig. 1a). High signal intensity in MRI components was shown in green by changing its pseudo coloring. (Fig. 1b).

### Evaluation of the images

**Fig. 2:**
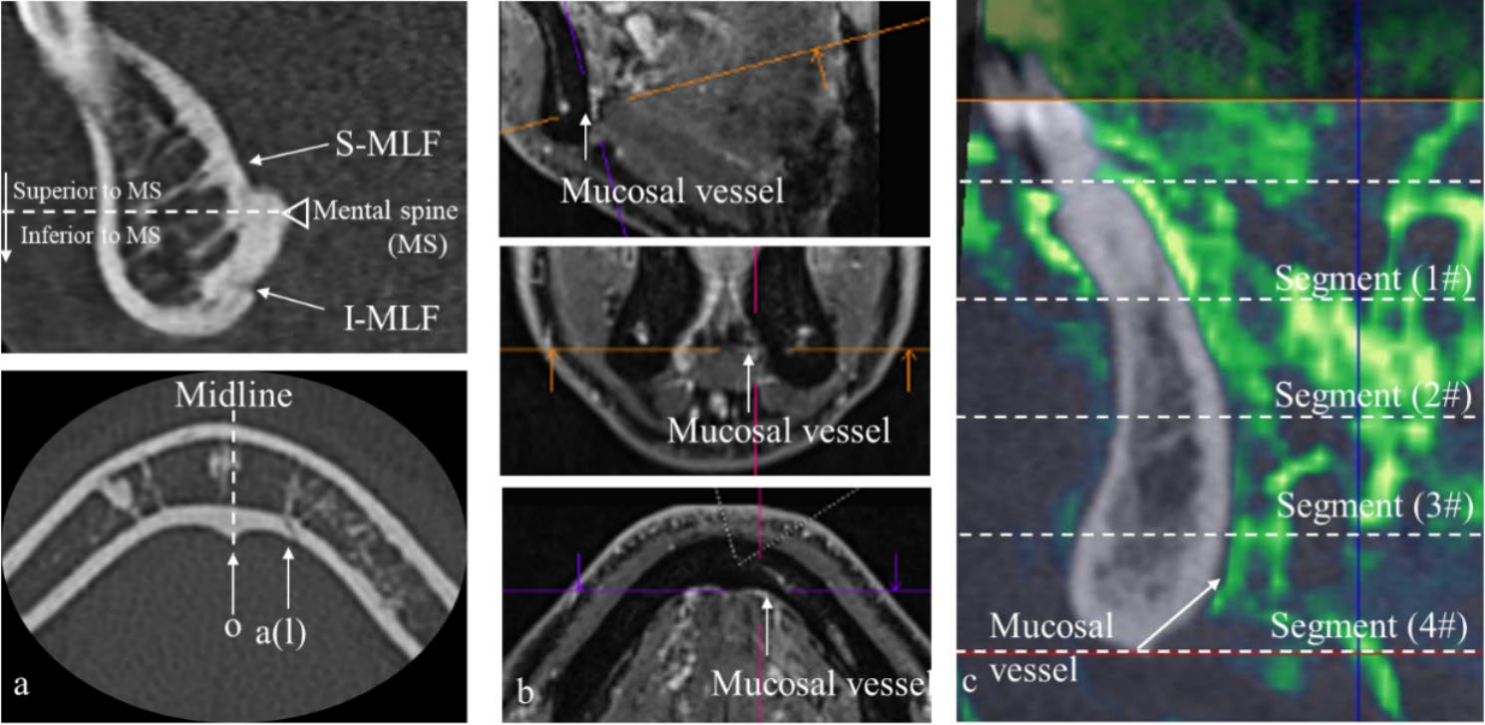
Evaluation of MLFs and LMVs **a**. Position of MLFs in relation to mental spine in CT (sagittal image, horizontal image respectively). MLFs: mandibular lingual foramens; S-MLF: superior to the mental spine on the midline; I-MLF: inferior to the mental spine on the midline; a(l): left side to the midline; o: on the mental spine **b**. LMVs in MRI (sagittal image, coronal image, horizontal image respectively).LMVs: lingual mucosal vessels. The lingual and facial vessels were searched in thier anatomic location firstly and then sublingual and submental vessels. When the high signal intensity of these veseels extended the surface of the mandibular lingual cortex, they were marked it as LMVs **c**. CT/MRI fusion volumetric image of mucosal vessels and tooth position (sagittal image). 4 average segments were divided from the alveolar bridge to the lowest point of the mandibular inferior border # refers to the segment of tooth

Taking the mental spine (MS) as the reference plane, the location of MLFs was divided into 5 positions (on the midline superior to the mental spine (s-MLF), on the midline inferior to the mental spine (I-MLF), left side to the midline (al), right side to the midline (ar) or on the mental spine(o)). (Fig. 2a). In MRI, we firstly focused on lingual and facial vessels in related anatomic location and then sublingual and submental vessels. When the high signal intensity on the surface of the mandibular lingual cortex was continuous with above vessels, we marked it as LMVs in relevant segment. (Fig. 2b). In order to simulate the clinical reality of dental implants, we divide each tooth position of the anterior mandibular portion from the alveolar bridge to the lowest point of the mandibular inferior border into 4 segments on average and get the final total of 40 regions. The location, number, diameter, and LMVs in the 40 regions were marked on the MRI and CT fusion images. (Fig. 2c).The measurements were taken by two investigators. The inter-observer reliability was highly satisfactory with intraclass correlation coefficient (ICC) greater than 0.92.

### Statistical analysis

The differences in the mean value of diameter were assessed by using Student’s t test while one-way ANOVA was used to reveal the difference in the distribution of MLFs in different regions for males and females. To get the correlation between MLFs and LMVs, Spearman’s correlation coefficient was used. And the Spearman’s r values of 0.40, 0.60, and 0.80 were regarded as weak, moderate, and strong associations, respectively. The plus and minus signs represent positive and negative correlations. The heatmap was run in R (R version R 3.4.1 and R Studio version 1.0.143). Analysis was performed using IBM Statistics SPSS 21.0 (SPSS, Chicago, IL, USA) and R, considering P < 0.05 statistically significant.

## Results

Twenty-six Japanese individuals including 12 males and 14 females participated in this study. All had at least two MLFs and one patient had six MLFs. 62 MLFs (73.81%) were located below the incisors while 18 MLFs (21.43%) were located below the premolars and only 4 MLFs (4.76%) were located below the canines.

The number and mean diameter of MLFs in males and females were discussed by MLFs location, and detailed data were shown in Table 1. It showed the distribution of the number of MLFs in each region, and one-way ANOVA was used and revealed no statistical difference in the distribution of the number of MLFs in different regions between males and females. Twelve males had a total of 50 MLFs with a mean diameter of 0.21 mm, and 14 females had a total of 34 MLFs with a mean diameter of 0.08 mm, with a female bias in mean diameter. There was a direct statistical difference between females and males inferior to the mental spine. (P < 0.05) (Table 1).

**Table 1:** The Location and diameter of MLFs in males and females

Location	Male		Female		P value	
	number	Diameter(mm)	Number	Diameter(mm)		
Superior	N = 15	0.19 ± 0.43	N = 11	0.08 ± 0.019		0.102
Inferior	N = 17	0.26 ± 0.53	N = 10	0.07 ± 0.015		0.021
a(l)	N = 13	0.21 ± 0.43	N = 8	0.09 ± 0.021		0.131
a(r)	N = 4	0.08 ± 0.01	N = 5	0.10 ± 0.033		0.253
o	N = 1	0.09	N = 0			
sums	N = 50	0.21 ± 0.44	N = 34	0.08 ± 0.022	0.794	0.001

MLFs: mandibular lingual foramens; a(l): left side to the midline; a(r): right side to the midline; o: on the mental spine

**Fig. 3:**
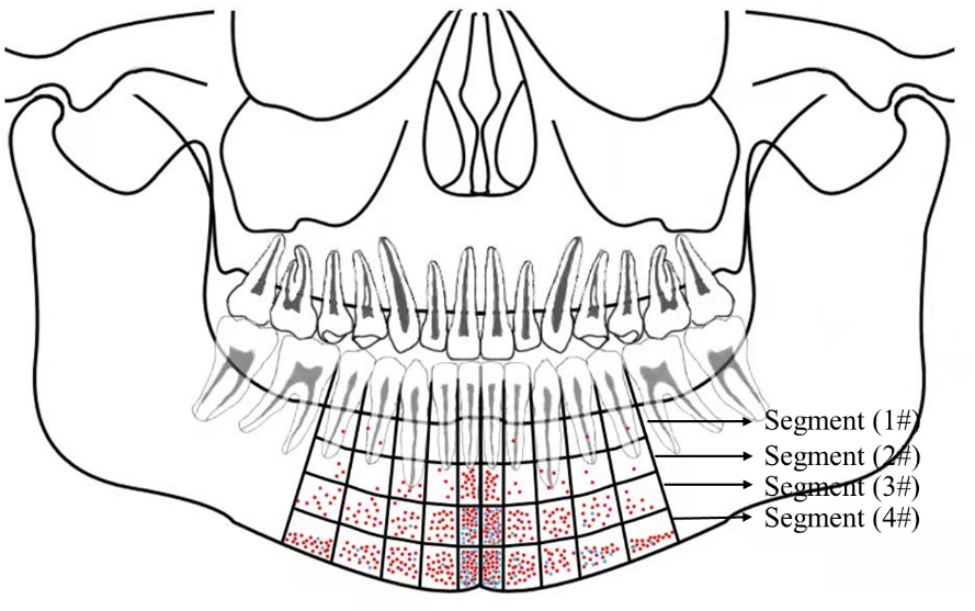
The distribution of MLFs and LMVs MLFs: mandibular lingual foramens;LMVs: lingual mucosal vessels # refers to the segment of the tooth, blue dots refer to MLFs; red dots refer to mucosal vessels

The greatest number of LMVs was distributed under the C1 and D1, while the number of them decreased to the right and left respectively. Among the four segments, LMVs were most distributed in the fourth segment, followed by the third segment, with the least presence of vessels in the first segment. (Fig. 3).

**Fig. 4:**
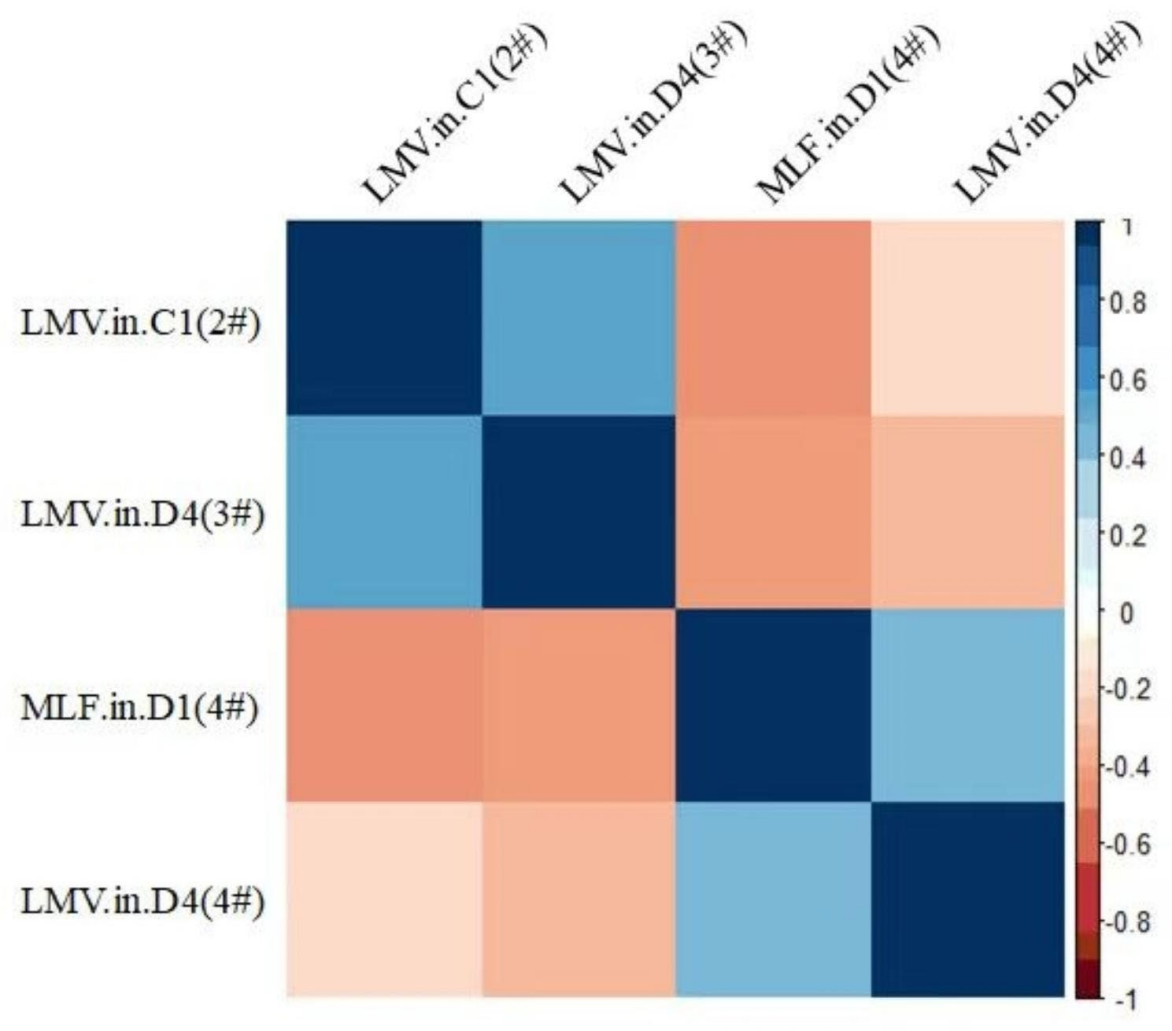
Heatmap of MLFs in D1 (4# ) with LMVs MLFs: mandibular lingual foramens; LMVs: lingual mucosal vessels # refers to the segment of the tooth, C1(2#) refers to the second segment of C1 tooth, and so on

Spearman’s correlation coefficient was used to analyze the distribution of LMVs and the location of MLFs in the mandibular. The MLFs in D1(3#) were negatively moderately correlated with the LMVs in the C1(2#). The MLFs in D1(4#) were negatively moderately correlated with the LMVs in C1(2#) and D4(3#) while it was positively moderately correlated with the LMVs in D4(4#).(Fig. 4.). The MLFs in C1(3#) showed a weak positive correlation with the LMVs in D4(3#). Detail data was shown in Table 2.

**Table 2:** The results from Spearman’s correlation coefficient

Location of MLFs	Location of mucosal vessels	Spearman’s r value	P value
D1(3#)	C1(2#)	-0.474^*^	0.014
D1(4#)	C1(2#)	-0.523^**^	0.006
	D4(3#)	-0.447^*^	0.022
	D4(4#)	0.451^*^	0.021
D2(3#)	C2(2#)	0.469^*^	0.016
	D3(2#)	0.410^*^	0.038
	D4(1#)	0.410^*^	0.038
D2(4#)	D1(3#)	-0.554^**^	0.003
	D3(2#)	0.435^*^	0.026
	D4(1#)	0.435^*^	0.026
	D5(1#)	0.554^**^	0.003
	C5(3#)	0.422^*^	0.032
D3(4#)	C5(1#)	0.693^**^	0.000
D4(3#)	D5(3#)	0.433^*^	0.027
	C4(1#)	0.458^*^	0.019
D5(4#)	D4(2#)	0.458^*^	0.019
C1(3#)	D4(3#)	0.393^*^	0.047
C2(4#)	C5(4#)	-0.554^**^	0.003
C3(4#)	C4(1#)	0.693^**^	0.000
C5(4#)	C4(1#)	0.458^*^	0.019
	D5(2#)	0.693^**^	0.000

*The correlation is significant at a confidence level (two-sided) of 0.05

**The correlation is significant at a confidence level (two-sided) of 0.01

The Spearman’s r values of 0.40, 0.60, and 0.80 were regarded as weak, moderate, and strong associations, respectively. The plus and minus signs represent positive and negative correlations

# refers to the segment of the tooth, D1(3#) refers to the third segment of D1 tooth, and so on

## Discussion

Although the anterior mandible was widely recognized as a relatively safe in dental implant, hemorrhage in tongue-floor resulting airway obstruction were the most dangerous complications. Some scholars suggested that MLFs were the potential anatomical factor [26]. Vessels of MLFs were tiny compared with the neurovascular bundle in mandibular canal and limited by the diameter of mandibular lingual canal. Meanwhile, more researchers supported that compared with the penetration of MLFs, unnecessary lingual cortical bone perforation and its resulting mucosal vessels damage took the primary responsibility [7, 19]. Other case reports evaluated patients’ situations and supported the conclusion as well [6, 8, 9, 14, 18, 20–22, 27]. As a result, we focused on mucosal vessels which may be involved after lingual cortex perforations. Although we could not distinguish them from vein and artery, both arterial and venous perforations are dangerous. Arterial bleeding is rapid, while venous is more dangerous. The slow bleeding is not easily treated on time after the patient leaving the clinic, which may result in airway obstruction and even death. Therefore, all mucosal vessels in the interforaminal region of the mandible in our study are included to get a better conclusion. Although the cadaver study could lead to more accurate conclusions about the vessels [30], it did not apply to clinical patients personalized information. C.Deepho applied CT/MRI fusion volumetric images to determine the mandible canal position in the mandible and found that the CT/MRI fusion volumetric images were more accurate compared to CT measurements alone [28]. In the present work, we have used the CT/MRI fusion volumetric images to evaluate the number, location, diameter of MLFs and the location of LMVs to get position relationship between them.

In the previous research, investigators mostly used CT to analyze the number and structure of MLFs [29]. Some researchers tried to use MRI to analyze the vascular and neural structures, but it’s poor in detecting MLFs [28, 30]. We used fusion volumetric images in 26 Japanese subjects. The results of MLFs were consistent with other studies, with the number of MLFs distributed between 2 and 6, most predominantly below the incisors, followed by the premolars and finally the canines [10, 11, 13, 29]. When we considered sex differences, we found similar findings to those of Yildirim [31] who state that women have slightly lower values in each of the variables analyzed. However, we only found one statistically significant difference in the inferior mental spine region. Furthermore, we found positional correlation between MLFs and LMVs, which was paid little attention in other researches. The anterior mandibular region was divided into 4 equal segments under each teeth to simulate the clinical reality of dental implants.The MLFs in fourth segment of D1 were positively moderately correlated with LMVs in fourth segment of D4, while the MLFs in third segment of C1 showed a weak positive correlation with LMVs in third segment of D4. Since CBCT or CT is still the modality used before clinical implantation, these results may offer a reference to dentists when only MLFs can been seen in image.

A limitation of this study is that there were few cases included. The detection of LMVs is highly dependent on the quality of the MRI image while artifacts have an impact on the results, and the patient is required to remain motionless in the body position, etc. Therefore, the number of patients we included in this retrospective study was small. However, the patients’ ages ranged from 11 to 81 years. The development of mandible and LMVs in circumpubertal growth and age-related alterations in old people were not in consideration for the small number of subjects. Further research should be undertaken to investigate the remaining alveolar bone remodeling. Additionally, MRI would not be necessary in clinical implant. The results of this study should be confirmed with further prospective studies.

In summary, our studies suggest that the location of MLFs is correlated with that of LMVs, which may demonstrate that there is a high or low likelihood of the presence of LMVs somewhere. The risk of bleeding from an inadvertent puncture of the lateral lingual bone plate may increase in LMVs area. Since MRI is not yet widely used in dental clinical examination and CT is limited in some parts, we hope to offer reference to dentists to indicate the possibility of LMVs by the location and number of the patient’s MLFs in CT images before dental implants, to obtain relatively safe and dangerous areas for implantation to reduce complications.

## Conclusion

In this study, CT/MRI fusion volumetric images are used to evaluate the anterior mandibular bone. The results indicate the correlation between MLFs and LMVs, which may offer reference to clinical dentists in the interforaminal region of dental implants.

## Data Availability

The datasets used and/or analyzed during the current study available from the corresponding author on reasonable request.
